# Single genetic locus improvement of iron, zinc and β-carotene content in rice grains

**DOI:** 10.1038/s41598-017-07198-5

**Published:** 2017-07-31

**Authors:** Simrat Pal Singh, Wilhelm Gruissem, Navreet K. Bhullar

**Affiliations:** Plant Biotechnology, Department of Biology, ETH Zurich, Zurich, Switzerland

## Abstract

Nearly half of the world’s population obtains its daily calories from rice grains, which lack or have insufficient levels of essential micronutrients. The deficiency of micronutrients vital for normal growth is a global health problem, and iron, zinc and vitamin A deficiencies are the most prevalent ones. We developed rice lines expressing Arabidopsis *NICOTIANAMINE SYNTHASE 1* (*AtNAS1*), bean *FERRITIN* (*PvFERRITIN*), bacterial *CAROTENE DESATURASE* (*CRTI*) and maize *PHYTOENE SYNTHASE* (*ZmPSY*) in a single genetic locus in order to increase iron, zinc and β-carotene content in the rice endosperm. NAS catalyzes the synthesis of nicotianamine (NA), which is a precursor of deoxymugeneic acid (DMA) iron and zinc chelators, and also chelate iron and zinc for long distance transport. FERRITIN provides efficient storage of up to 4500 iron ions. PSY catalyzes the conversion of GGDP to phytoene, and CRTI performs the function of desaturases required for the synthesis of β-carotene from phytoene. All transgenic rice lines have significantly increased β-carotene, iron, and zinc content in the polished rice grains. Our results establish a proof-of-concept for multi-nutrient enrichment of rice grains from a single genetic locus, thus offering a sustainable and effective approach to address different micronutrient deficiencies at once.

## Introduction

Deficiencies of minerals and essential vitamins, collectively termed “hidden hunger”, are prevalent in human populations all over the world. Iron deficiency anemia (IDA), zinc deficiency and vitamin A deficiency (VAD) are among the most common forms of micronutrient deficiencies, which are especially widespread in the developing world. Around two billion people are affected by iron deficiency worldwide, in both developed and developing countries^1^. IDA leads to retarded mental development, decreased immunity, and higher maternal and perinatal mortality. Maternal and neonatal mortality due to IDA are significant contributors to global mortality rate, especially in the developing world alone^2^. Globally, IDA affects 43% children as well as 38% pregnant and 29% non-pregnant women^3^. Similarly, zinc deficiency is also prevalent globally, and based on severity it is categorized into severe, moderate, and mild forms. The most common symptoms of zinc deficiency include male hypogonadism, retarded growth, cell-mediated immune dysfunction and abnormal neurosensory changes^4^. VAD is also a major health concern and causes night blindness and xeropthalmia. It is the main cause of preventable childhood blindness and a major contributor to morbidity and mortality from infections in children and pregnant women^5^. Around 250 million preschool children suffer vitamin A deficiency and are at a higher risk of death as a result of measles, diarrhea and malaria. On a yearly basis, between 250,000 to 500,000 vitamin A-deficient children become blind, with half of them dying within a year of losing their vision^6^.

Various epidemiological surveys conducted in developing countries showed that the prevalence of iron deficiency anemia is higher in populations affected by vitamin A deficiency^7^. The occurrence of micronutrient deficiencies has also been associated with the monotonous, cereal-based diets of the affected populations^8^. Various intervention strategies such as diet diversification, supplementation, and fortification are being utilized to combat micronutrient deficiencies in these populations. Addressing the deficiencies through diet diversification requires the consumption of micronutrient-rich food, which still is difficult in developing countries due to poverty and thus the lack of access to diverse foods. Similarly, providing vitamin A and iron supplements to an affected population is a useful short-term intervention, but often it is not sustainable because it requires stable government policies, appropriate social infrastructure, and continuous investment^9^. Thus, the direct improvement of nutritional quality of staple foods by biofortification offers an effective solution to address micronutrient deficiencies^10^. Rice (*Oryza sativa* L.) is a major contributor to the human diet, with half of the world’s population depending on it to meet their daily caloric requirements^11^. However, this staple cereal lacks β-carotene (provitamin A) and has insufficient iron, zinc and other nutrients in the endosperm (polished grains) to meet dietary requirements. Biofortification of rice endosperm for essential nutrients can have a significant positive impact on global human health. In order to provide 30% of dietary estimated average requirement (EAR) of iron, 15 μg/g DW iron should be present in polished rice grains, while 28 μg/g DW of zinc can provide 40% of the EAR^12^. Earlier studies estimated that 17 μg/g DW β-carotene should be present in polished rice grains to provide 50% of the EAR^12^. More recently, De Moura *et al*.^13^ determined that biofortified rice containing 8–12 μg/g β-carotene, when widely adopted and substituted for white rice, is an effective method of reducing the population prevalence of vitamin A deficiencies. Based on data from Bangladesh, Indonesia and Philippines, they conclude that biofortified rice containing more than 12 μg/g β-carotene had no added advantage over a higher substitution rate (around 70%) of biofortified rice with white rice^13^. The breeding efforts over the past several years have failed to achieve the recommended iron content in the rice endosperm, mainly due to a lack of genetic diversity^14^. Similarly, rice cultivars accumulating β-carotene in the endosperm could not be identified in germplasm screenings^15^. Genetic engineering, on the other hand, has been successfully used to enrich the rice endosperm with iron, provitamin A, and folate^16–19^.

Iron is required for key physiological and metabolic activities of plants, and hence biofortification for iron requires a careful choice of strategy^20^. So far, successful iron biofortification has involved a combination of two approaches: iron uptake and translocation, and iron storage. The first approach is the enhancement of iron uptake from the soil, and then its translocation within the plant. Rice, a graminaceous plant, also utilizes a chelation-based strategy (Strategy II) for iron acquisition, in addition to direct uptake of Fe^2+^. In strategy II, plants release mugineic acid (MA) family phytosiderophores (PS) into the rhizosphere that bind Fe^3+^ in form of strong hexadentate chelates, and these chelates are transported into the roots by special transporters^21, 22^. One such PS is deoxymugineic acid (DMA), which is synthesized from S-adenosyl-L-methionine in a reaction catalyzed by NICOTIANAMINE SYNTHASE (NAS), NICOTIANAMINE AMINOTRANSFERASE (NAAT), and DEOXYMUGINEIC ACID SYNTHASE (DMAS)^23^. In this pathway, NAS is the key enzyme for synthesis of nicotianamine (NA), the precursor of DMA. Constitutive overexpression of *NAS* increased the iron content in polished rice grains of transgenic plants by 2- to 4-fold^24–26^. The second aspect of increasing endosperm iron content involves FERRITIN, an iron storage complex of 24 protein subunits arranged to form a hollow structure. A single ferritin complex is capable of storing up to 4,500 ferric molecules in a bioavailable form^27^. The endosperm-specific expression of *FERRITIN* increased the iron content in polished rice by 2- to 3.7-fold^28–32^. The combined expression of bean *FERRITIN* (*PvFERRITIN*) and Arabidopsis *NAS1* (*AtNAS1*) increased iron content in the rice endosperm by 6-fold and zinc content by 1.3-fold^18^. More recently, rice lines expressing a combination of soybean *FERRITIN* and rice *NAS2* were reported to have a 7.5-fold increase in iron content and 3.3-fold increase in zinc content in the polished grains^17^. In addition to the increased iron content, the overexpression of *NAS* often results in beneficial increases of the zinc content, which are likely due to the increased production of PS because zinc also complexes with PS^33^.

The rice endosperm does not produce β-carotene, which is the precursor of vitamin A. However, the immature rice endosperm can synthesize geranylgeranyl diphosphate (GGDP), the precursor molecule required for the synthesis of β-carotene. Production of β-carotene in plants requires PHYTOENE SYNTHASE (PSY) and three additional enzymes: PHYTOENE DESATURASE, ζ-CAROTENE DESATURASE and LYCOPENE β-CYCLASE. However, CAROTENE DESATURASE (CRTI) of *Pantoea ananatis* (formerly *Erwinina uredovora*) performs the function of both the desaturases and cyclase^34^. Thus, the expression of *PSY* from daffodil and *PaCRTI* in rice endosperm resulted in the synthesis of β-carotene (Golden rice^34^). Paine *et al*.^19^ later reported that endosperm-specific expression of maize *PSY* (*ZmPSY*) and *PaCRTI* increased β-carotene in the rice endosperm by 23-fold compared to the golden rice prototype. Since the concepts of rice endosperm biofortification with high β-carotene as well as iron and zinc content are now well established independently, the next challenge is to combine these traits as a single locus to facilitate breeding of multi-nutrient rice varieties.

In this study, we transformed rice with Arabidopsis *NAS1* (*AtNAS1*) expressed under the control of the constitutive *CaMV 35S* promoter, bean *FERRITIN* (*PvFERRITIN*) expressed under the control of the endosperm-specific rice *GLOBULIN* (*OsGLOBULIN*) promoter, and *PaCRTI* and *ZmPSY* under the expression of the endosperm-specific rice *GLUTELIN1* (*OsGLUTELIN1*) promoter in a single construct. We also transformed the high iron NFP rice^18^ with a construct carrying *ZmPSY* and *PaCRTI*. In either case, iron, zinc and β-carotene levels are significantly increased in the rice endosperm. This establishes a proof of concept for adapting the biofortification approaches to simultaneously address several important micronutrient deficiencies.

## Results

### β-carotene enrichment of high iron- and zinc-containing endosperm of NFP rice grains

**Figure 1:**
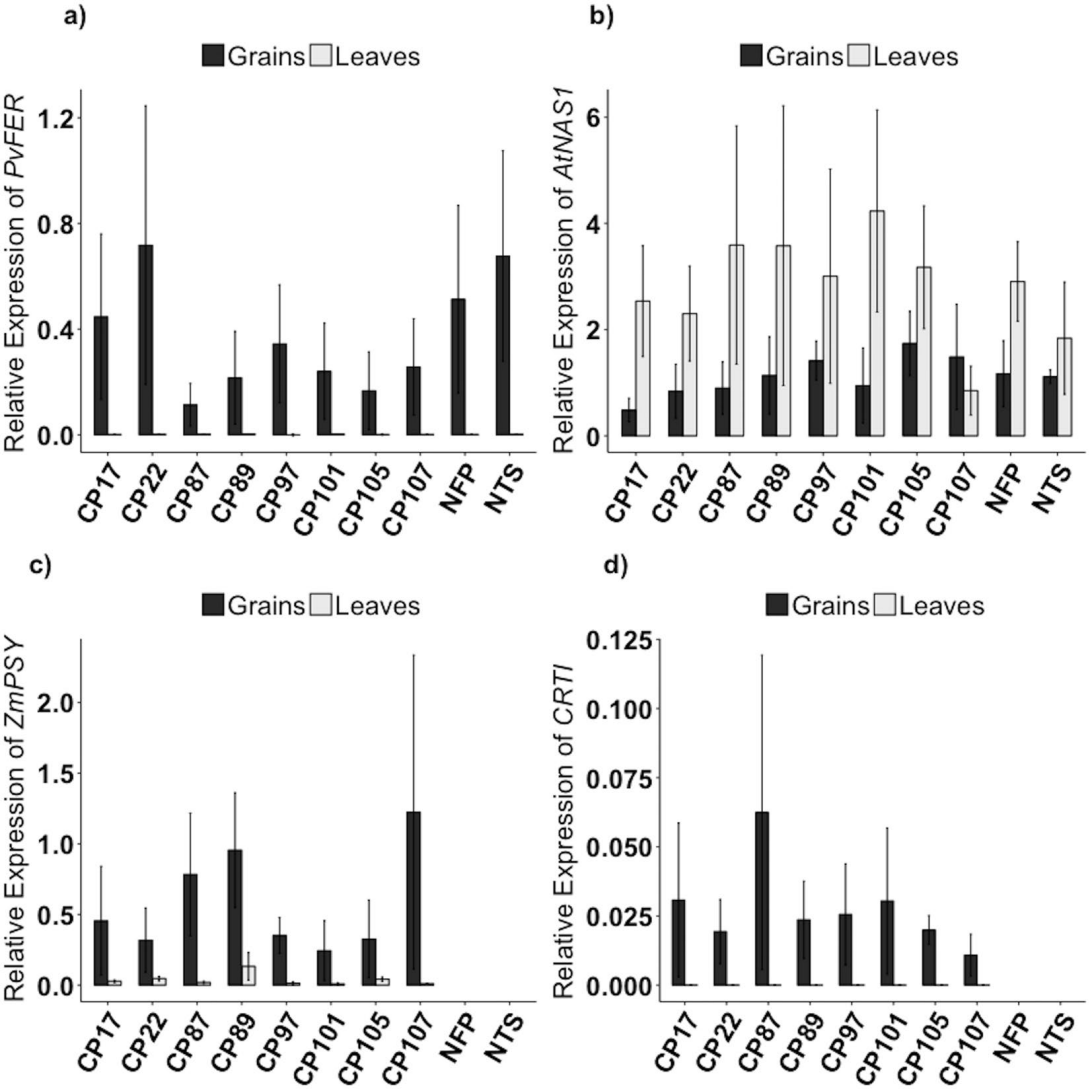
Transgene expression of the CP lines. Relative expression of *PvFERRITIN*, *AtNAS1*, *PaCRTI*, and *ZmPSY* transgenes in T2-generation CP lines at 18 d after flowering (DAF). The data was normalized with the endogenous expression of *Os16530*. NTS is the segregating NFP sibling that does not contain the *PaCRTI*-*ZmPSY* construct. Values are the average of three biological replicates (±SD) for all except NTS, for which two biological replicates were used.

**Figure 2:**
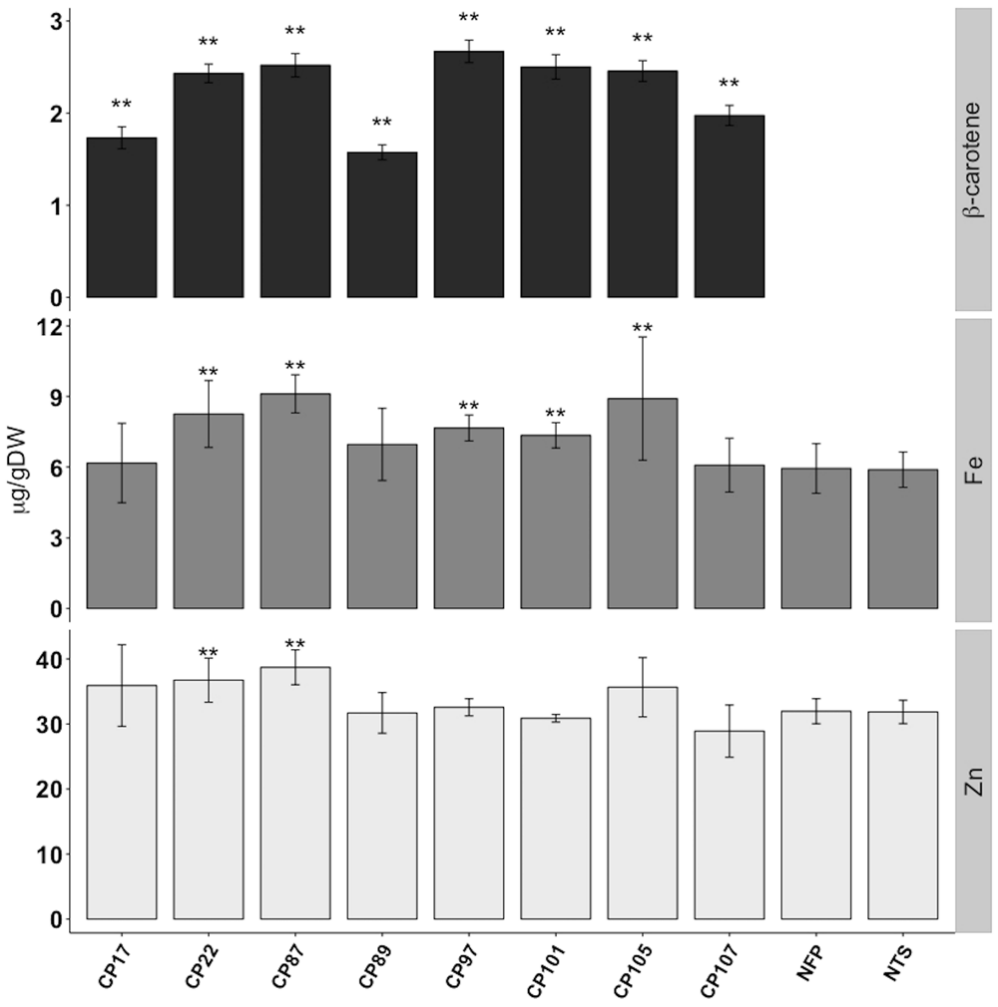
β-carotene and metal content of CP lines. β-carotene, iron (Fe) and zinc (Zn) content in polished grains of T3 CP lines. Values are the mean of three biological replicates (±SD). Black asterisks above the bars indicate statistically significant differences calculated using Student’s T test in comparison to the control line NFP (*P < 0.05; **P < 0.01). NTS is the segregating NFP sibling that does not contain the *PaCRTI*-*ZmPSY* construct.

**Figure 3:**
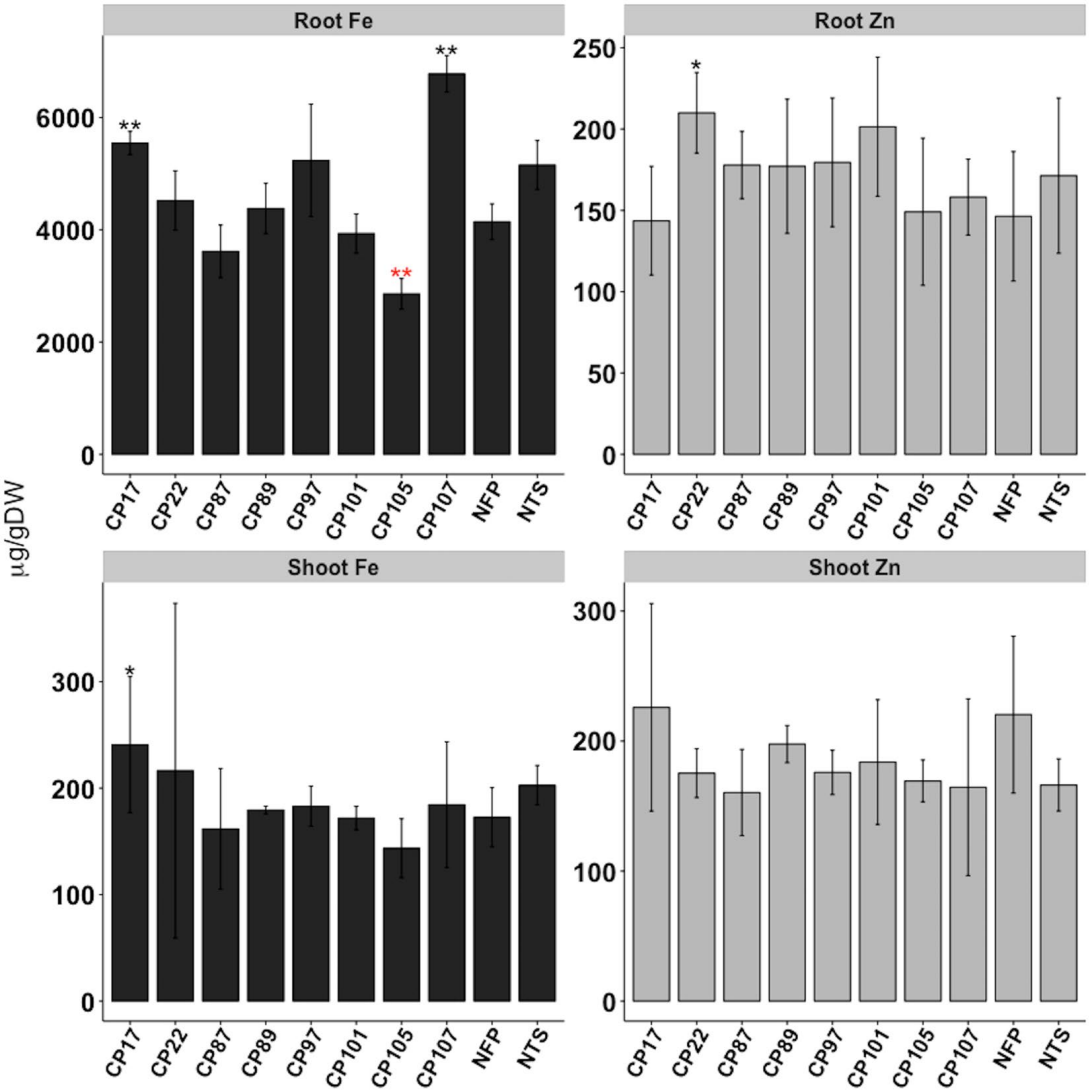
Metal content in roots and shoots of CP lines. Iron (Fe) and zinc (Zn) in the shoots and the roots of 18 d seedlings of T3 CP lines. Values are the mean of three biological replicates (±SD). Black and red asterisks above the bars indicate statistically higher and lower significant differences calculated using Student’s T test, respectively, in comparison to the control line NFP (*P < 0.05; **P < 0.01). NTS is the segregating NFP sibling that does not contain the *PaCRTI*-*ZmPSY* construct.

In order to assess if increased iron and β-carotene synthesis can be combined in rice endosperm, the high-iron NFP rice^18^ expressing *AtNAS1* and *PvFERRITIN* was super-transformed with a construct containing *PaCRTI* and *ZmPSY* (CP lines). Eight CP lines with single insertions of the construct (Supplementary Fig. S1) were analyzed for transgene expression and for metal as well as β-carotene content in the endosperm. As expected, *PvFERRITIN*, *PaCRTI*, and *ZmPSY* transgene expression was restricted to the grains of these lines, and no expression was found in the leaves (Fig. 1). *AtNAS1* was expressed in both grains and leaves of the CP lines, similar to that in the control line NFP. All transformed lines produced β-carotene in the endosperm. The β-carotene content in polished T3 grains ranged from 1.57 to 2.69 μg/g DW with highest content in CP97, while polished grains of control NFP produced no β-carotene (Fig. 2). Furthermore, the iron content in the T3 polished grains ranged from 6.1 to 9.1 μg/g DW, as compared to 5.9 μg/g DW in the NFP control line (Fig. 2). Five lines CP22, CP87, CP97, CP101, and CP105 showed further increases of 1.2- to 1.5-fold in the endosperm iron content as compared to NFP, with highest iron levels in CP87. Zinc content was only increased in the polished grains of CP22 (36.7 μg/g) and CP87 (38.7 μg/g), representing a change of 1.1- and 1.2-fold as compared to the NFP control (32 μg/g), respectively. Overall, line CP87 had superior iron, zinc and β-carotene levels in the polished grains, while CP105 still had significantly improved levels of iron and β-carotene. Additionally, the copper content in most of the lines was significantly increased, except in lines CP17, CP101, and CP105 (Supplementary Fig. S2). Manganese content decreased in the polished grains of lines CP22, CP87, CP97, and CP101, while magnesium content in lines CP22, CP87, CP97, and CP105 increased by 1.3 to 1.5-fold (Supplementary Fig. S2). Iron, zinc, copper, manganese, and magnesium contents in the shoots and the roots of the transformed lines were not altered, except for few differences as compared to the control (Fig. 3 and Supplementary Fig. S3). Phenotypic characterization of T2-generation transgenic plants grown in soil showed some variability for growth parameters including days to flowering, plant height, 1000 grain weight, while tiller number did not differ as compared to the control (Supplementary Table S1).

### Simultaneous enrichment of rice endosperm with β-carotene, iron and zinc by expression of *PvFERRITIN, AtNAS1, ZmPSY* and *PaCRTI* from a single construct

**Figure 4:**
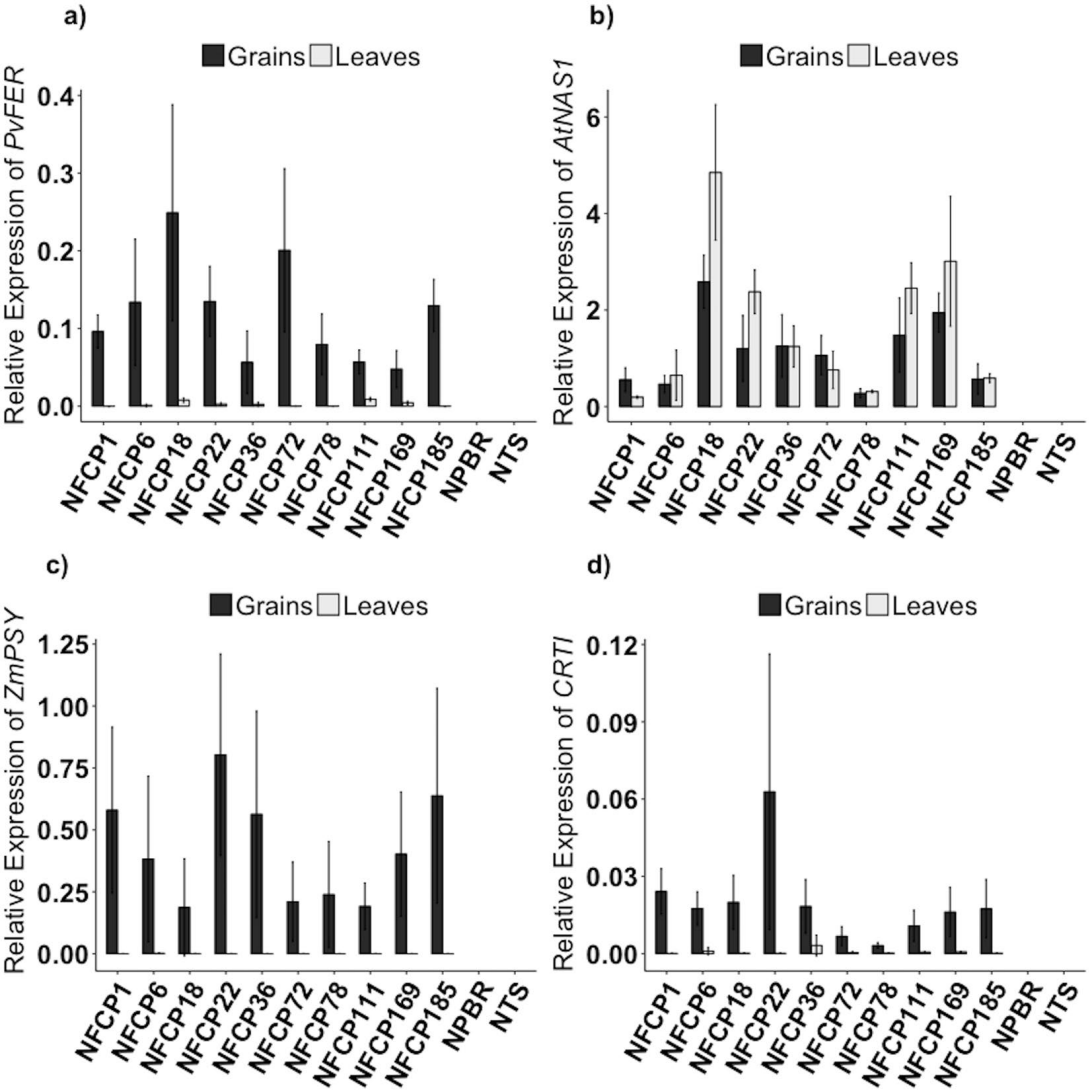
Transgene expression of the NFCP lines. Relative expression of transgenes *PvFERRITIN*, *AtNAS1*, *PaCRTI*, and *ZmPSY* in T2 NFCP lines at 18 d after flowering **a)** Relative expression of *PvFERRITIN*. **b)** Relative expression of *AtNAS1*. **c)** Relative expression of *ZmPSY*. **d)** Relative expression of *PaCRTI*. No expression of transgenes was observed in Nipponbare (NPBR) control and NTS. The data was normalized with the endogenous expression of *Os16530*. Values are the average of three biological replicates (±SD).

**Figure 5:**
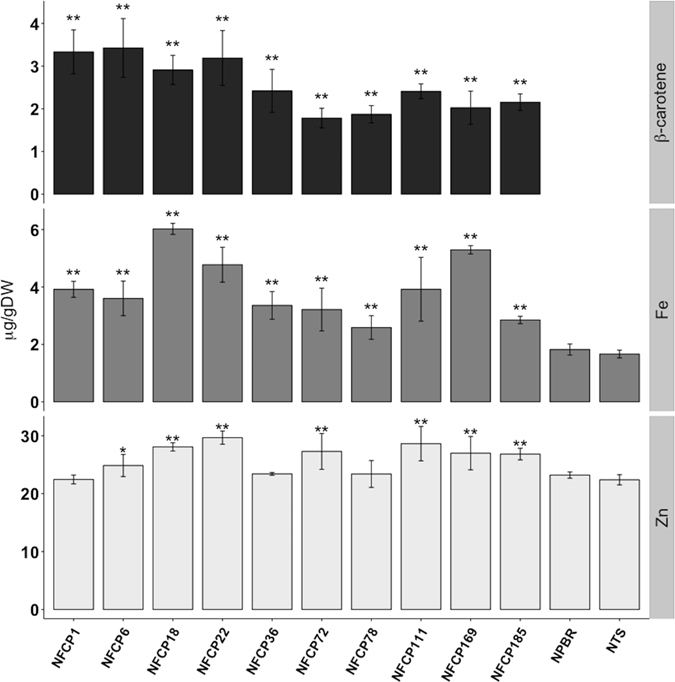
β-carotene and metal content of NFCP lines. β-carotene, iron (Fe) and zinc (Zn) content in polished grains of T3 NFCP lines. Values are the mean of three biological replicates (±SD). Asterisks above the bars indicate statistically significant differences calculated using Student’s T test, in comparison to the control line Nipponbare (NPBR) (*P < 0.05; **P < 0.01). NTS is the non-transgenic sibling.

Ten T2 NFCP lines expressing *PvFERRITIN, AtNAS1, ZmPSY* and *PaCRTI* in *O. sativa* japonica cv. Nipponbare from a single construct and with a single insertion of the construct (Supplementary Fig.S4) were selected for advanced analysis. All of the lines showed expression of *PvFERRITIN*, *PaCRTI*, and *ZmPSY* in the grains, while no significant expression was detected in the leaves (Supplementary Table S3 and Fig. 4). Expression of *AtNAS1* was found in both grains and leaves of the transgenic lines as expected. In comparison to the non-transgenic sibling and Nipponbare controls, all transformed lines showed significantly higher iron content in the polished grains and with exception of three lines, the zinc content was also increased. Additionally, β-carotene was produced in the polished grains of all lines (Fig. 5). The endosperm iron content of the transgenic lines ranged from 2.6 to 6.02 μg/g DW as compared to 1.82 μg/g DW in Nipponbare. Among all lines, NFCP18 had the highest iron content (6.02 μg/g), which is an increase of 3.3-fold as compared to the control grains. NFCP22 and NFCP169 also showed an iron increase of more than 2.5-fold in the polished grains. The β-carotene content in the polished grains (Fig. 5) ranged between 1.9 and 3.4 μg/g DW, while no β-carotene was detected in the polished grains of the control lines. The β-carotene content was highest in line NFCP6 with 3.4 μg/g DW, and NFCP18 (line with highest iron content) had 2.1 μg/g DW of β-carotene in its endosperm. The zinc content of the transgenic lines ranged from 24.9 to 29.7 μg/g DW, which represents an increase of 1.07- to 1.28-fold as compared to the Nipponbare control (23.2 μg/g DW). Overall, lines NFCP18 and NFCP22 have the most favorable combination of increased iron, zinc and β-carotene content in the endosperm. The divalent metals including copper, magnesium, and manganese were also increased in most of the lines (Supplementary Fig. S6). Significantly higher copper content, with 1.06- to 1.41-fold increases, was found in most of the lines, except NFCP111, NFCP169, and NFCP185. NFCP78 was an exception, and showed a decrease in copper content (3.5 μg/g) as compared to the control line (4.6 μg/g DW). The manganese content ranged from 4.9 to 8 μg/g DW and thus increased by 1.2- to 1.8-fold as compared to the control, with the exception of lines NFCP78 and NFCP185. Similarly, magnesium content was increased in most of the transgenic lines as compared to the control, except for lines NFCP78 and NFCP185 (Supplementary Fig. S6).

**Figure 6:**
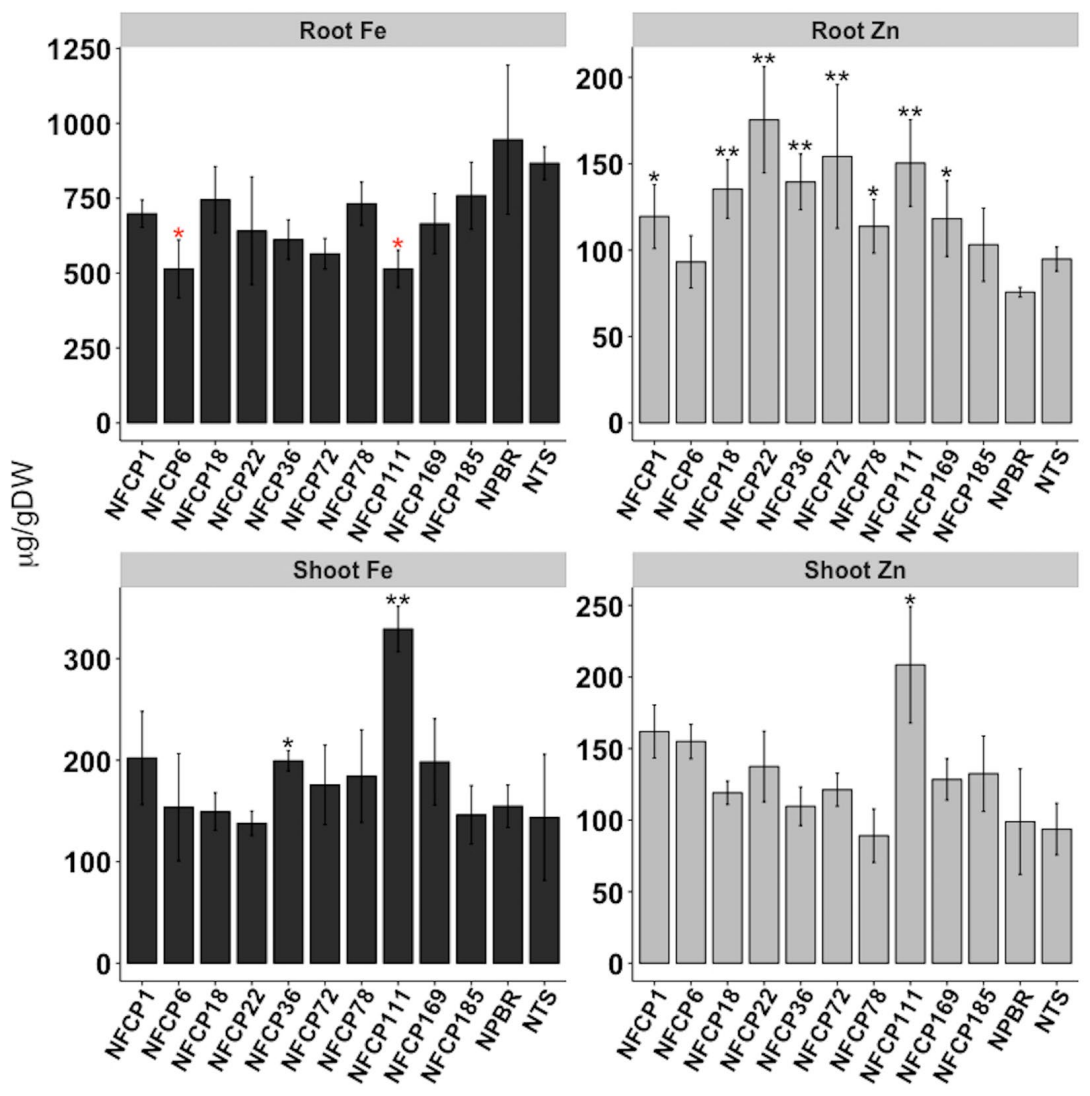
Metal content in roots and shoots of NFCP lines. Iron (Fe) and zinc (Zn) in the shoots and the roots of 18 d-old seedlings of T3 NFCP lines. Values are the mean of three biological replicates (±SD). Black and red asterisks above the bars indicate statistically higher and lower significant differences calculated using Student’s T test, respectively, in comparison to the control line Nipponbare (NPBR) (*P < 0.05; **P < 0.01). NTS is the non-transgenic sibling.

With some exceptions, the metal profiles in the shoots and the roots of the NFCP lines were comparable to the control lines (Fig. 6 and Supplementary Fig. S7). The iron content in the shoots and roots of the NFCP lines was comparable to the Nipponbare control, except for slightly increased iron in the shoots of lines NFCP36 and NFCP111, and a decrease in root iron levels in NFCP6 and NFCP111 (Fig. 6). Although zinc content in the NFCP shoots was not different from the control (except NFCP111), the zinc content in the roots was significantly higher in most NFCP lines, except NFCP6 and NFCP185 (Fig. 6). The concentration of copper, manganese, and magnesium was not changed in shoots and roots of most of the transformed lines, indicating that with the exception of root zinc levels the overall metal homeostasis of the NFCP plants is not altered (Supplementary Fig. S7). Phenotypic characterization of T2-generation transgenic plants grown in soil showed some variability for measured parameters such as days to flowering, plant height, tiller, 1000 grain weight (Supplementary Table S2).

## Discussion

In developing countries, hidden hunger is often associated with the dependence of affected populations on monotonous cereal-based diets. The causes for this over-reliance on starchy cereals, especially in developing countries, are numerous and complex. Low incomes, a lack of access, poor healthcare, and cultural traditions all contribute to a lack of balanced diets. In this regard, increasing one or more micronutrients in staple food crops have substantial potential in addressing micronutrient deficiencies. The biofortification of rice with micronutrients such as iron, zinc, vitamin A and folate has been successful in recent years, demonstrating that this approach is feasible. For example, the development of Golden Rice (first in 2000 and improved in 2005) proved that it is possible to successfully engineer the β-carotene pathway in rice endosperm^19, 34^. Several studies reported similar success in developing rice lines with high iron content in the endosperm^17, 18, 25, 28, 35^. However, these improvements of individual micronutrients only address a single deficiency at a time. The combination of several nutritional traits into a single variety could offer significant benefits to human health. For example, rice lines with improved iron and β-carotene levels in the endosperm could address two most detrimental nutritional deficiencies, especially since the prevalence of IDA is higher in populations suffering from VAD^7^.

We demonstrate that rice can be engineered with genes facilitating an increase in iron and zinc content as well as the production of β-carotene in the endosperm, either as two traits expressed from independent loci or as two traits combined in a single genetic locus. Since the NFCP lines express *AtNAS1*, *PvFERRITIN*, *PaCRTI* and *ZmPSY* from a single locus, increased uptake and translocation of iron and zinc as well as the targeted storage of iron and production of β-carotene in the endosperm could be expected but needed to be validated to demonstrate synergistic function of the four genes. The NFCP lines have up to 3.3-fold iron and 1.28-fold zinc increases in the endosperm, respectively, and also produce β-carotene at significantly higher levels compared to the control plants. Similar β-carotene levels were obtained in the NFP lines transformed with *PaCRTI* and *ZmPSY*, while the iron content in these lines (CP) was even higher than in the control NFP lines. The iron and zinc increases are consistent with the activity of NAS in increasing the production of nicotianamine (NA) and deoxymugineic acid (DMA), which facilitate uptake and transport of iron and zinc^21, 22, 36^. The endosperm of wild type rice plants does not produce β-carotene, but synthesizes the GGDP precursor. The production of β-carotene in the NFCP rice endosperm is therefore solely due to the activity of PSY (which converts GGDP to phytoene) and CRTI (which synthesizes β-carotene from phytoene) and consistent with the results first obtained for Golden Rice^34^.

The amount of β-carotene produced in NFCP lines as compared to the improved Golden Rice version 2^19^ is, however, lower, with a maximum of 3.4 μg/g DW. Several factors, including the different genetic background of the rice cultivar and the small number of transgenic lines we used in our proof-of-concept study, could account for the difference. Several hundred independent transgenic lines were generated during the production of Golden Rice 2 that contained between 9 and 37 μg/g of carotenoids, with 31 μg/g of β-carotene content in the line with highest levels of carotenoids^37^. Our work demonstrates the feasibility of combining β-carotene, high-iron and zinc traits in a single cultivar. Furthermore, a recent study reported that substituting 70% of white rice intake with biofortified rice containing 4 μg/g β-carotene could reduce the prevalence of inadequate vitamin A intake by 23% in Indonesia and Philippines, and by 42% in Bangladeshi women^13^. Thus, line NFCP18 could already provide useful β-carotene supplementation as well as 40% of the recommended increase of iron content and 100% of the zinc requirements. IDA and VAD co-occur within populations, and β-carotene has been suggested to facilitate iron mobilization and transport, and enhance overall iron absorption^38, 39^. We therefore expect that combining high-iron with β-carotene in rice endosperm could benefit iron absorption in the human gut, but this needs to be tested in nutritional studies. Furthermore, the increased iron, zinc and β-carotene levels in the NFCP lines address three micronutrient deficiencies at once. The effectiveness of combined supplementation of iron, vitamin A, and zinc in micronutrient-deficient populations has been well documented in different studies^39^. Combining different nutritional traits as a single genetic locus in a major staple crop is a significant step towards realizing combined supplementation for health benefits, although further improvements and optimization of the combined traits are still possible. Selected lines must now be tested in confined field trials to demonstrate trait performance and stability. If successful, the multi-nutrient trait locus can be transferred to farmer-preferred rice cultivars or rice mega-varieties, either by breeding or direct transformation. Selected best performing lines would then need to be fully characterized before approval by regulatory authorities. Best performing lines combining several nutrient traits into a single genetic locus allows breeders to easily introduce these into breeding lines. Moreover, engineering a multi-nutrient trait using proven genes in single and simple inserts may be favorable from a regulatory point of view. Together, we demonstrate that it is possible to combine nutritional traits in a staple crop, which is a promising approach to greatly improve the nutritional quality of agricultural crops.

## Materials and Methods

### Transformation vectors

The construct expressing *PvFERRITIN* under the control of the *OsGLOBULIN* endosperm-specific promoter and *AtNAS1* under the control of constitutive *CaMV 35S* promoter was previously described^18^. The *KpnI* restriction site in the construct (located outside of the coding region) was mutated in order to use *KpnI* for further cloning. The *PvFERRITIN* and *AtNAS1* containing fragment was excised using *BamHI* and *PstI*. The pCAMBIA-1300 binary vector was the final destination vector used for transformation. The *HYGROMYCIN PHOSPHOTRANSFERASE* (*HPT*) selection marker in pCAMBIA-1300 was replaced with *PHOSPHOMANNOSE ISOMERASE* (*PMI*) using the restriction site *XhoI*, thus generating pCAMBIA-1300PMI. The *PvFERRITIN* and *AtNAS* fragment was cloned into pCAMBIA-1300PMI using the *BamHI* and *PstI* sites, generating pCAMBIA-1300PMI-NASFER. The *ZmPSY* (NM_001114652) under the control of the *OsGLUTELIN* endosperm-specific promoter (D00584 1568-2406) along with the nopaline synthase gene terminator (nosT) was synthesized using GenScript^®^ (http://www.genscript.com) in the pUC57 plasmid, generating pUC57-GtPsy. *KpnI* and *XbaI* restriction sites were incorporated in the synthesized fragment for cloning purposes. Similarly, the DNA sequence for the pea RUBISCO SSU transit peptide (X00806) combined with *PaCRTI* and nosT was synthesized with GenScript^®^ (http://www.genscript.com) in pUC57, named as pUC57-ssuCrt. Afterwards, the *OsGLUTELIN* promoter was inserted upstream of pUC57-ssuCrt using the restriction sites *KpnI* and *XbaI*, generating pUC57-GtssuCrt. Later, both constructs were combined in the pBluescript II SK(-) vector using the restriction site *KpnI* flanking the 5’ and 3’ end of the gene cassette, generating the pBluescript II SK(-) PsyCrt construct. In the last step, *ZmPSY* and *CRTI* were cloned into the pCAMBIA-1300PMI-NASFER and pCAMBIA-1300 binary vectors using *KpnI* to generate the pCAMBIA-1300PMI-NASFER-CRTPSY and pCAMBIA-1300-CRTPSY constructs, respectively. The generated constructs were verified both by sequencing and restriction digestion.

### Rice transformation, genotypic characterization, and greenhouse conditions

The *Oryza sativa* (rice) cultivar Nipponbare (*japonica* type) was transformed with pCAMBIA-1300PMI-NASFER-CRTPSY construct generating transgenic lines, hereafter named NFCP. The high-iron NFP rice^18^ expressing *AtNAS1* under the control of the *CaMV 35S* promoter and *PvFERRITIN* and *Aspergillus fumigates PHYTASE* (*AfPHYTASE*) genes under the control of the *OsGLUTELIN* promoter, was transformed with pCAMBIA-1300-CRTPSY construct generating transgenic lines, hereafter named CP. The *Agrobacterium tumefaciens* strain EHA105 was used for rice transformation. Transformation, and regeneration of NFCP lines were performed as described by Nishimura *et al*.^40^ while selection for *PMI* was done on mannose-containing media as described by Lucca *et al*.^41^. For generation of CP lines, transformation, selection and plant regeneration was performed as described by Nishimura, *et al*.^40^ and *HPT* selection was done on hygromycin-containing media. The plant growth chamber conditions were maintained at 16 h light/8 h dark, 28 °C and 60% humidity. Genomic deoxyribonucleic acid (DNA) isolation from three-week-old seedlings in the T0, T1, and T2 generations was performed as previously described^42^. PCR was used to confirm full-length integration of the constructs and subsequent selection of transgenic lines was done in T0, T1 and T2 generations with transgene specific primers (Supplementary Table S3). Southern blot hybridization using digoxigenin (DIG) labeling was performed on transgenic lines, with *PmlI* digested genomic DNA for NFCP, and *HindIII* digested genomic DNA for CP, to select for transformants with a single insertions of the constructs. A *PMI* specific probe was used for NFCP lines and a *HPT* specific probe was used for CP lines. For NFCP, 196 lines were generated of which 47 were identified as single copy insertions, but 27 lines showed full integration of the construct. For CP construct, 128 lines were generated of which 38 were identified as single copy insertions and 24 lines showed full integration of the construct. Based on preliminary nutrient data on T1 and T2 grains, 10 best NFCP lines and 8 CP lines were selected for further analysis (presented data). Selected transgenic lines were grown in commercial soil (Klasmann-Deilmann GmbH, Germany) in the greenhouse with conditions set at 12 h light, 30 °C, 80% humidity and 12 h dark, 22 °C, 60% humidity.

### Metal ion measurements

Plants were harvested at maturity, and spikes were dried at 37 °C for three days. Grain samples were de-husked to obtain rice grains and polished with a grain polisher (Kett grain polisher ‘Pearlerst’, Kett Electric Laboratory, Tokyo, Japan) for one minute. For metal quantification in the shoots and the roots, 18 days old seedlings (T_3_ generation) were grown in hydroponic solution containing 0.70 mM K_2_SO_4_, 2 mM Ca(NO_3_)_2_, 0.1 mM KH_2_PO_4_, 0.5 mM MgSO_4_, 0.1 mM KCl, 10 μM H_3_BO_3_, 0.5 μM MnSO_4_, 0.2 μM CuSO_4_, 0.01 μM (NH_4_)_6_Mo_7_O_24_, 0.5 μM ZnSO_4_, 100 μM Fe-EDTA and pH of 5.5. Both shoot and root samples were dried at 60 °C for five days. 200 mg of sample for polished grains and 20 mg of sample for shoots and roots was boiled in 15 ml of 65% v/v HNO_3_ solution at 120 °C for 90 min. Three ml of 30% v/v H_2_O_2_ was added and boiled at 120 °C for 90 min. The National Institute of Standards and Technology (NIST) rice flour standard 1568a (NIST, USA: https://www-s.nist.gov/srmors/view_detail.cfm?srm=1568a) was treated and analyzed in the same manner and used as a quality control for every measurement. Metal concentrations were determined using inductively coupled plasma-optical emission spectroscopy (ICP-OES) (Varian Vista-MPX CCD Simultaneous ICP-OES). The wavelength used for iron, zinc, copper, manganese, and magnesium was 238.204, 213.857, 324.754, 257.610, and 285.213, respectively. The data were analyzed using the Student’s t-test to determine statistically significant differences among the tested lines.

### β-carotene measurement

Grains were processed for β-carotene measurements after 7-9 weeks of harvest. Polished grains were homogenized to a fine powder and a one-gram sample was dissolved in two ml of distilled water by sonication and vortexing. In order to evaluate the efficacy of extraction, a control sample with known amount of β-carotene was prepared. Rehydrated samples were incubated at 60 °C for 10 min, followed by centrifugation for 10 min at 3300 rpm, and the supernatant was collected in a tube. Extraction was repeated three times with one ml of acetone, each time collecting the supernatant for final pooling. Carotenoids extraction was done twice from the pooled sample with 2 ml of 2:1 v/v PE:DE (petroleum ether:diethylether). The samples were mixed by inverting and vortexing, followed by centrifugation for five min at 3300 rpm, and the supernatant was collected. Later, the collected supernatant was dried with a stream of nitrogen gas and then was re-dissolved in 1.5 ml of acetone. β-carotene standards with concentration of 1 μg/ml, 2 μg/ml, 3 μg/ml, 4 μg/ml, 6 μg/ml, and 8 μg/ml were prepared for the calibration curve. Measurement of β-carotene was done using a VARIAN UV-VISIBLE Spectrophotometer at 454.9 nm (n = 3). β-carotene content in the test samples was calculated based on the calibration curve in comparison to the known standard concentration^43^.

### Quantitative real-time PCR

Quantitative real-time PCR (qRT-PCR) was performed to analyze the expression of the transgenes. Total RNA was extracted from grains and leaves collected 18 days after flowering from T2-generation plants. Grain RNA was extracted as described by Singh *et al*.^44^, and leaf RNA was extracted using Trizol® reagent (Invitrogen, USA). RNA was treated with DNase I (Thermo Fisher Scientific, Inc., USA) to eliminate genomic DNA. The RevertAid^TM^ first strand cDNA synthesis kit (Thermo Fisher Scientific, Inc., USA) was used for cDNA synthesis. qRT-PCR was performed on the LightCycler® 480 Instrument (Roche, Switzerland). The total reaction volume of 10 μl included 1 μl cDNA, 0.2 μl forward primer, 0.2 μl reverse primer, 5 μl Sybrgreen Mastermix (Applied Biosystems, Ltd., USA), and 3.6 μl H_2_O. Primers were designed using a CLC genomics workbench (Supplementary Table S3). The Ct value was obtained from LightCycler® 480 Instrument (Roche, Switzerland). *Os01g0147200* encoding IWS1, C-terminal family protein was used as a reference gene for data normalization. The data normalization was done as described by Liu *et al*.^45^.

## Electronic supplementary material

Supplementary Information
