# Supplementary material for: Single genetic locus improvement of iron, zinc and β-carotene content in rice grains
Source: Sci Rep. 2017 Jul 31;7:6883. doi: 10.1038/s41598-017-07198-5 (PMC5537418; doi:10.1038/s41598-017-07198-5)
Supplement: Supplementary file 1 — Supplementary Information [file 41598_2017_7198_MOESM1_ESM.pdf]

Single genetic locus improvement of iron, zinc and β-carotene content in rice grains

Simrat Pal Singh, Wilhelm Gruissem, and Navreet K. Bhullar*

Plant Biotechnology, Department of Biology, ETH Zurich, Zurich, Switzerland

***Correspondence**

Dr. Navreet K. Bhullar

Plant Biotechnology,

Department of Biology

ETH Zurich

Universitaetsstrasse 2

8092 Zurich, Switzerland

bhullarn@ethz.ch

Supplementary Figure S1. Southern blotting of the CP lines

Example of Southern hybridization analysis of CP lines. Genomic DNA was digested by *HindIII*. Line CP17, CP22, CP87, CP89, CP97, CP101, CP105, and CP 107 were detected to contain single copy of transgene insertion and were chosen for further analysis. The Southern hybridization for line CP87 is repeated for clarity. NFP is the negative control and showed no signal for the *HPT* specific probe.

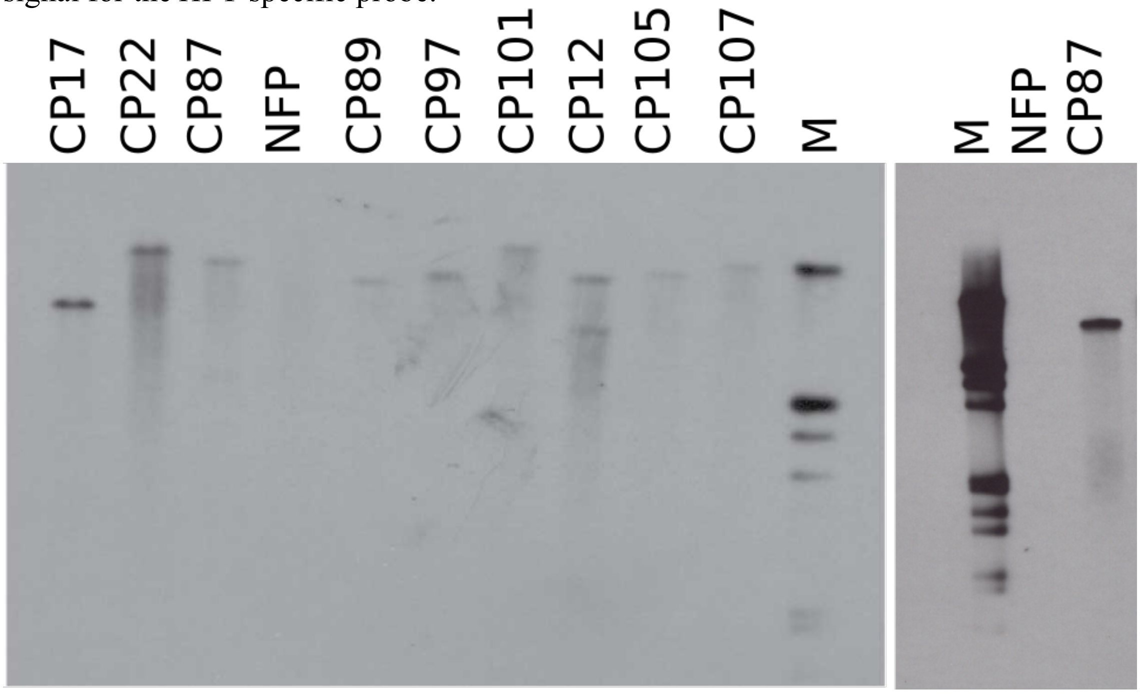

Supplementary Figure S2. Metal content of CP lines
Metal content in polished grains of T3 CP lines. Values are the mean of three biological replicates (±SD). Black and red asterisks above the bars indicate statistically higher and lower significant differences calculated using Student's T test, respectively, in comparison to the control line NFP (*P < 0.05; **P < 0.01). NTS is the segregating NFP sibling that does not contain the *PaCRTI-ZmPSY* construct.

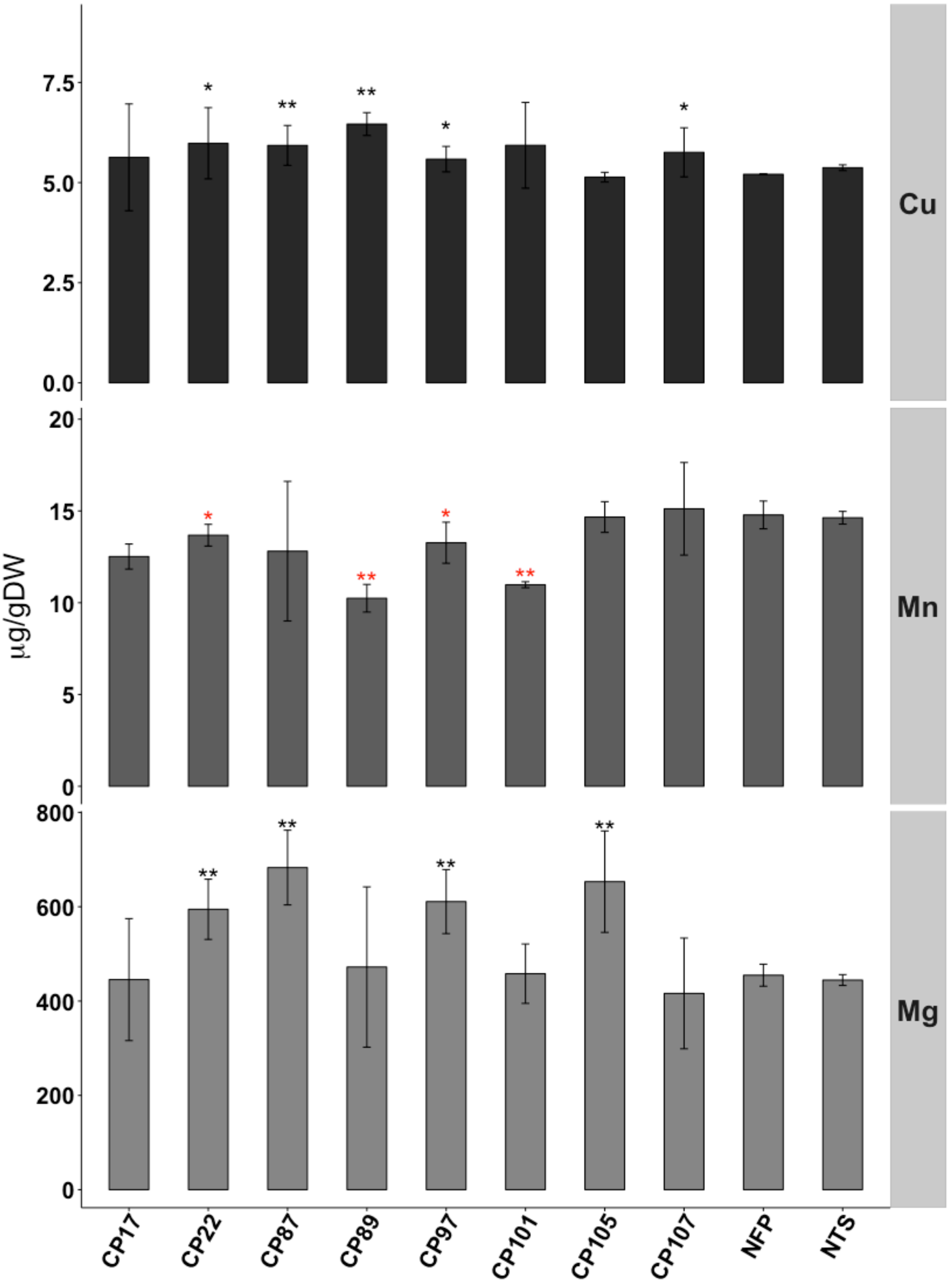

Supplementary Figure S3. Metal content of root and shoot of CP lines

Metal content in the shoots and the roots of 18 d seedlings of T3 CP lines. Values are the mean of three biological replicates (±SD). Black asterisks above the bars indicate statistically significant differences calculated using Student's T test, respectively, in comparison to the control line NFP (*P < 0.05; **P < 0.01). NTS is the segregating NFP sibling that does not contain the *PaCRTI-ZmPSY* construct.

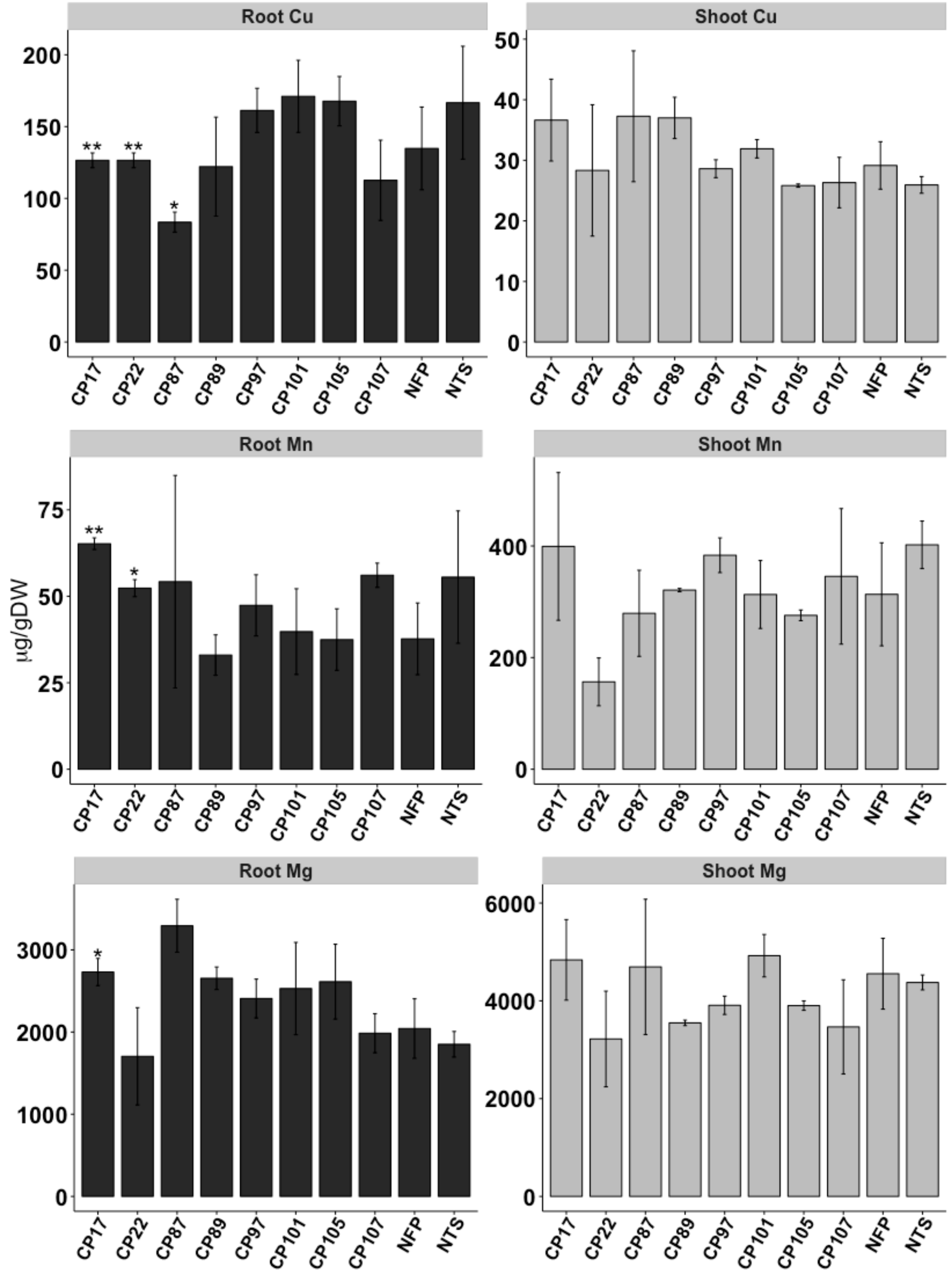

Supplementary Figure S4. Southern blotting of the NFCP lines
Example of Southern hybridization analysis of NFCP lines. Genomic DNA was digested by *PmlI*. Line NFCP1, NFCP6, NFCP18, NFCP22, NFCP36, NFCP72, NFCP78, NFCP111, NFCP169, and NFCP 185 were detected to contain single copy of transgene insertion and were chosen for further analysis. NPBR (Nipponbare) is the negative control and showed no signal for the *PMI*-specific probe.

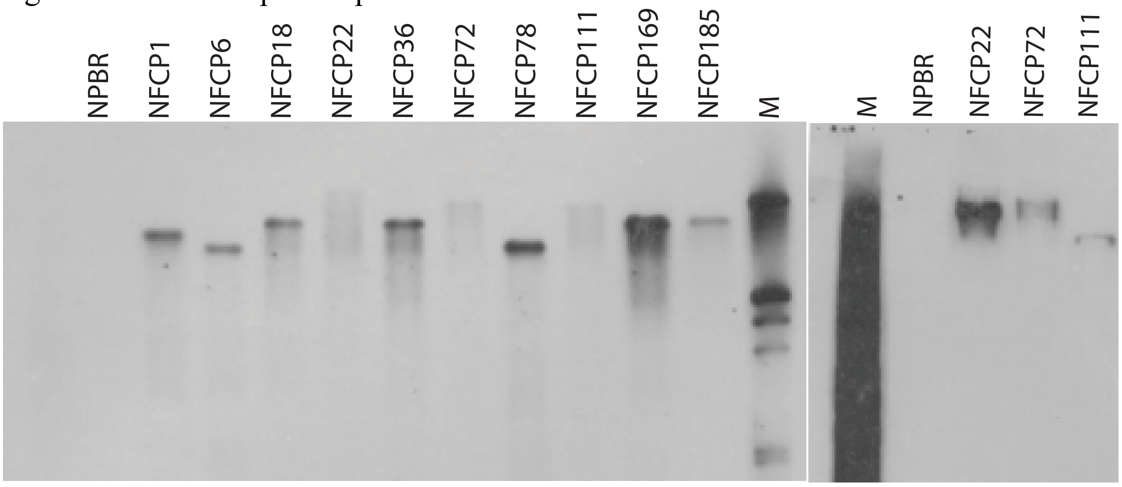

Supplementary Figure S5. PCR confirming transgene full length integration
Example of the PCR analysis of NFCP lines. Figure show the amplification of the PCR product for NFCP1 and NFCP 72 for **a)** LB-PMI **b)** PMI-CRT **c)** CRT-PSY **d)** PSY-35s **e)** 35s-AtNAS **f)** AtNAS-FER and **g)** FER-RB. NPBR is the negative control and showed no amplification. WC is the water control used in the reaction.

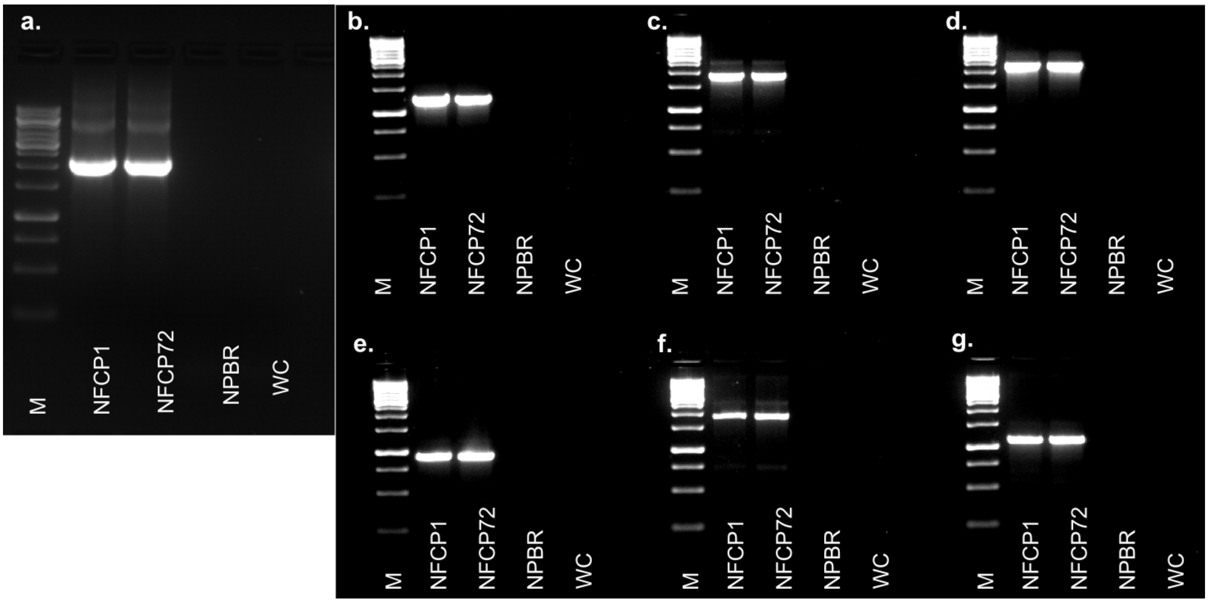

Supplementary Figure S6. Metal content of the NFCP lines

Metal content in content in polished grains of T3 NFCP lines. Values are the mean of three biological replicates (±SD). Black and red asterisks above the bars indicate statistically higher and lower significant differences calculated using Student's T test, respectively, in comparison to the control line Nipponbare (NPBR) (*P < 0.05; **P < 0.01). NTS is the non-transgenic sibling.

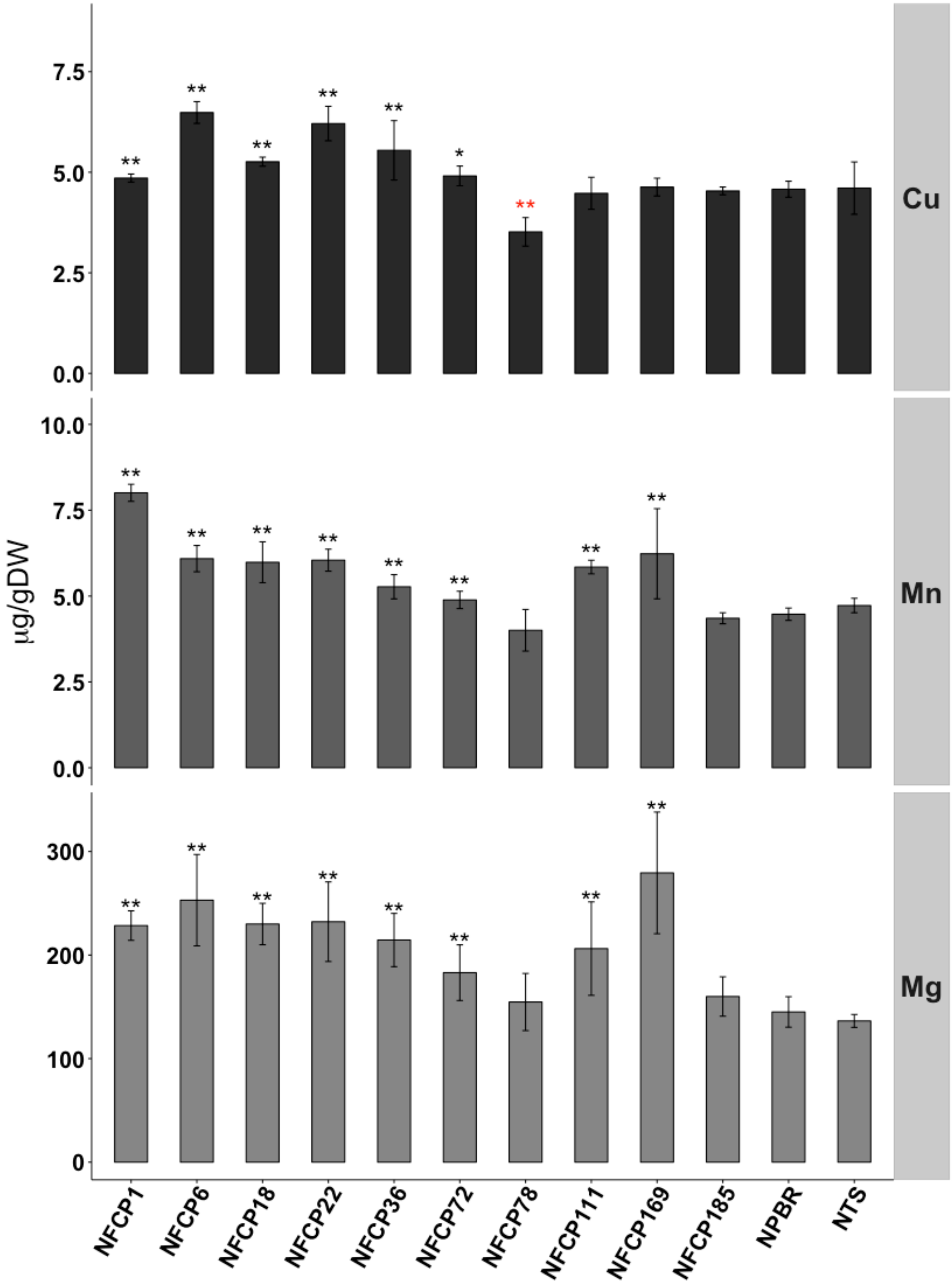

Supplementary Figure S7. Metal content of root and shoot of NFCP lines
Metal content in the shoots and the roots of 18 d seedlings of T3 NFCP lines. Values are the mean of three biological replicates (±SD). Asterisks above the bars indicate statistically higher significant values calculated using Student's T test, in comparison to the control line Nipponbare (NPBR) (*P < 0.05; **P < 0.01). NTS is the non-transgenic sibling.

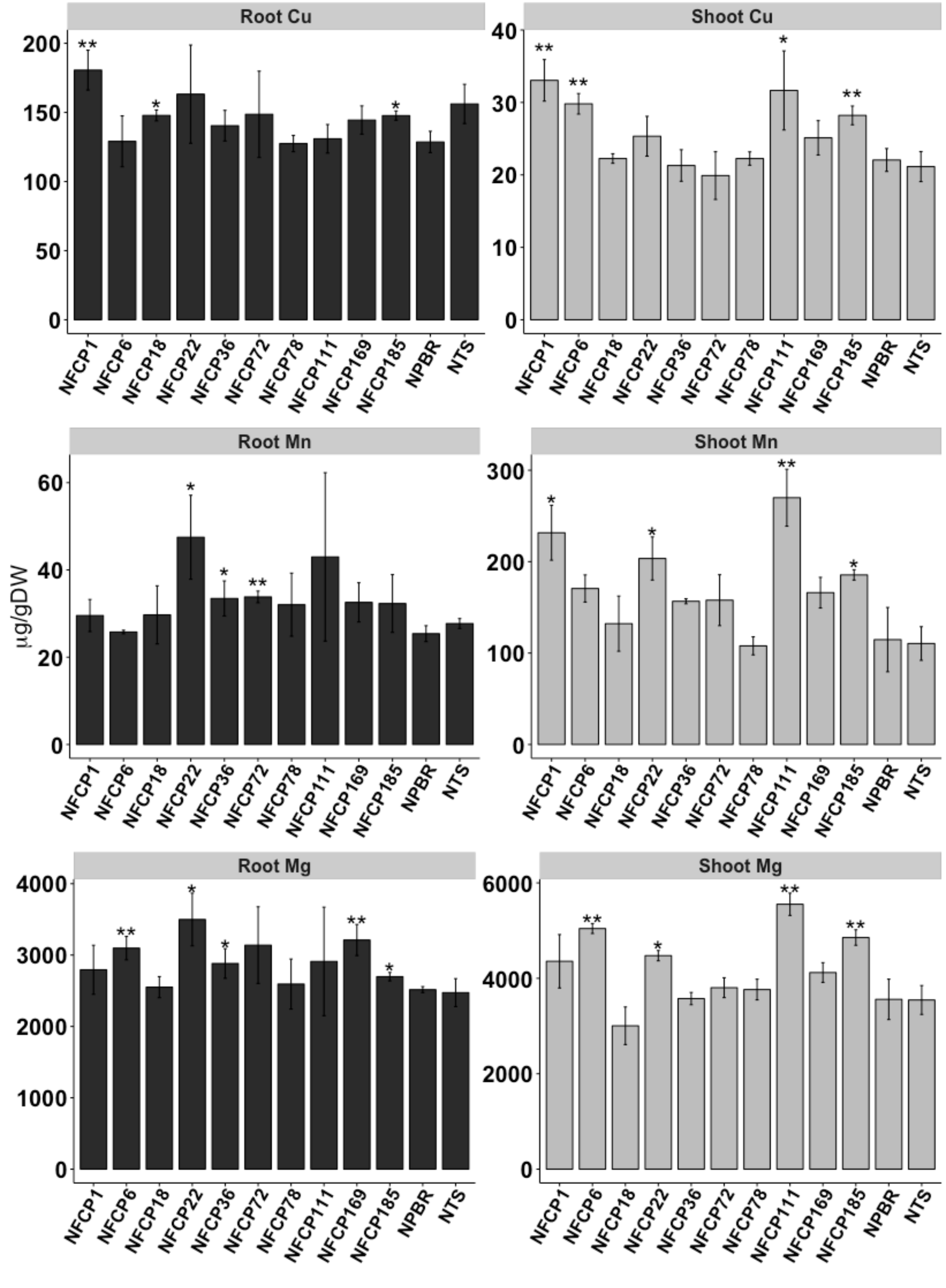

Supplementary table S1. Phenotypic performance of CP lines in the greenhouse
Phenotypic performance of T2 generation CP transgenic lines in the greenhouse. Values are the average of three biological replicates (± standard deviation). Transgenic plants were compared to Nipponbare. NTS is the segregating NFP sibling that does not contain the *PaCRTI-ZmPSY* construct. Black and red asterisks indicate statistically higher and lower significant values calculated using Student's T test, respectively (*P < 0.05; **P < 0.01).

Plant line	Days to flowering	Height(cm)	1000GW(g)	Tiller nr.	Yield per plant (g)
CP17	93.3 ± 1.2	69.1 ± 3.3,*	21.2 ± 0.3,**	9 ± 4	2.3 ± 0.8
CP22	93.7 ± 0.6	65.6 ± 3.1,*	21.3 ± 1	7 ± 2	3.7 ± 1.1
CP87	94.7 ± 1.2	69.2 ± 2.3,*	21.4 ± 2.1	8 ±2	3.1 ± 0.6
CP89	101.3 ± 0.6,**	62.5 ± 6.8	20.1 ± 0.9,*	6	3.4 ± 0.7
CP97	97.3 ± 3.1	69.2 ± 4.8,*	18.4 ± 0.1,**	7 ± 1	2.9 ± 0.2, *
CP101	101.7 ± 0.6,**	58.1 ± 12.9	21.1 ± 0.4,**	5 ± 1	3 ± 0.8
CP105	101.3 ± 0.6,**	63.7 ± 3.9,*	19.8 ± 3.5	5 ± 1	3.1 ± 0.7
CP107	104.3 ± 1.2,**	59.9 ± 1.9	19.9 ± 0.5,**	4 ± 1	3.6 ± 0.5
NFP	95.7 ± 1.2	78.4 ± 2.6	22.5 ± 0.2	5 ± 1	3.7 ± 0.4
NTS	91.3 ±0.6,**	83.6 ± 6.7	20.3 ± 0.8,*	5 ± 1	3.3 ± 0.5

Supplementary table S2. Phenotypic performance of NFCP lines in the greenhouse
Phenotypic performance of T2 generation NFCP transgenic lines in the greenhouse. Values are the average of three biological replicates (± standard deviation). Transgenic plants were compared to Nipponbare. NTS is non transgenic sibling. Black and red asterisks indicate statistically higher and lower significant values calculated using Student's T test, respectively (*P < 0.05; **P < 0.01).

Plant line	Days to flowering	Height(cm)	1000GW(g)	Tiller nr.	Yield per plant (g)
**NFCP1**	118 ± 1,**	57.3 ± 1	18.2 ± 0.6,**	11 ± 3,*	3.1 ± 0.8
**NFCP6**	115± 1,**	60.8 ± 0.7,**	17.6 ± 0.4,**	14 ± 2,*	3.7 ± 0.4
**NFCP18**	124 ± 1	56.1 ± 0.6,*	18.6 ± 0.3,**	10 ± 1,*	3.6 ± 0.3
**NFCP22**	124 ± 1	54.4 ± 2.2	18 ± 0.1,**	5 ± 1	2.4 ± 0.3
**NFCP36**	122*	56.9 ± 2.2	17.9 ± 0.4,**	10 ± 1,*	3.4 ± 1.1
**NFCP72**	121 ± 1.2,*	51.9 ± 1.4,**	20.2 ± 0.7	6 ± 1	3.2 ± 0.6
**NFCP78**	117 ± 2.9,*	53.1 ± 1.3,**	19.2 ± 0.3,*	9 ± 2	2.9 ± 0.8
**NFCP111**	120 ± 0.6,*	51.5 ± 1,**	18.5 ± 0.9,*	10 ± 3	3.3 ± 0.5
**NFCP169**	126 ± 1.2,*	48.7 ± 2.5,**	18.6 ± 0.8,*	6 ± 1	3 ± 0.8
**NFCP185**	120 ± 1.7,*	52.3 ± 1.2,**	18.2 ± 0.2,**	7 ± 2	3 ± 0.3
**Nipponbare**	123 ± 0.6	57.8 ± 0.5	20.7 ± 0.5	7 ± 2	3.6 ± 0.8
**NTS**	119 ± 0.6,**	52.6 ± 1.9,**	21.2 ± 0.3	6 ± 2	3.3 ± 0.5

Supplementary table S3. List of the primers used

Primers used for PCR screening, generation of probe for Southern hybridization, and for quantitative gene expression analysis (qRT-PCR)

Gene cassette	Forward primer	Reverse primer	Size (bp)
**PCR and Southern blot probe**			
PMI	CTGGCTAATGGTGGTTTCT	CGTGATGTGATTGAGAGT	837
HPT	CAAGCTGCATCATCGAAATTG	TCTGATCGAAAAGTTCGACAG	822
**qRT-PCR**			
PvFERRITIN	AAGCAGGAACCTTGGTGT	AGGGTACATTCTTGATCG	365
AtNAS1	GCACTTGGAGAAACACATGG	TCTGAGAGCATGAGCACTCC	64
PaCRTI	GGTGGCGAAGGGATTGC	ATCTGCGCCAGGCGTTT	59
ZmPSY	TGGTGTAATGTAGTTGGCGT	GGCGAGATCTGTGAGGAGT	150
Os01g0147200	AGCAGCTGAAAGCACCAAA	CACGCCCTTCAACACTGAG	63
**Fragment Integration PCR**			
LB-PMI	GGGGGATCTGGATTTTAGT	TGCCAGCTGCATTAATGA	2216
PMI-CRT	TGTTGTGTGGAATTGTGAG	GAAACGACAGGAAACAAGA	1220
CRT-PSY	TTGTAGACGAATTGCCAG	GGTAAAGGGAAGAAGTTG	1860
PSY-35s	TCTTATCTGTTCTGTGCCT	GTTCTGTTAGGTCCTCTATT	2279
35s-AtNAS	AACAGAACTCGCCGTAAA	TACGATGGATGTGAGAGG	944
AtNAS-FER	CCTCTCACATCCATCGTATT	ACCCAGAAAAGGGGGAAA	1877
FER-RB	AGGAGGTTAAGAAGGAAGAG	AAGGCGATTAAGTTGGGT	1147
